# Association of sex-specific abdominal adipose tissue with WHO/ISUP grade in clear cell renal cell carcinoma

**DOI:** 10.1186/s13244-023-01494-7

**Published:** 2023-11-19

**Authors:** Shichao Li, Zhouyan Liao, Kangwen He, Yaqi Shen, Shan Hu, Zhen Li

**Affiliations:** 1 Department of Radiology, Tongji Hospital, Tongji Medical College, Huazhong University of Science and Technology, Wuhan, Hubei China; 2 The Second School of Clinical Medicine, Southern Medical University, Guangzhou, China; 3 Department of Radiology, Guangdong Provincial People’s Hospital, Guangdong Academy of Medical Sciences, Guangzhou, Guangdong China

**Keywords:** Clear cell renal cell carcinoma, Tomography (X-ray computed), Obesity, Abdominal, Tumor grading

## Abstract

**Objectives:**

To explore the association between computed tomography (CT)-measured sex-specific abdominal adipose tissue and the pathological grade of clear cell renal cell carcinoma (ccRCC).

**Methods:**

This retrospective study comprised 560 patients (394 males and 166 females) with pathologically proven ccRCC (467 low- and 93 high-grade). Abdominal CT images were used to assess the adipose tissue in the subcutaneous, visceral, and intermuscular regions. Subcutaneous fat index (SFI), visceral fat index (VFI), intermuscular fat index (IFI), total fat index (TFI), and relative visceral adipose tissue (rVAT) were calculated. Univariate and multivariate logistic regression analyses were performed according to sex to identify the associations between fat-related parameters and pathological grade.

**Results:**

IFI was significantly higher in high-grade ccRCC patients than in low-grade patients for both men and women. For male patients with high-grade tumors, the SFI, VFI, TFI, and rVAT were significantly lower, but not for female patients. In both univariate and multivariate studies, the IFI continued to be a reliable and independent predictor of high-grade ccRCC, regardless of sex.

**Conclusions:**

Intermuscular fat index proved to be a valuable biomarker for the pathological grade of ccRCC and could be used as a reliable independent predictor of high-grade ccRCC for both males and females.

**Critical relevance statement:**

Sex-specific fat adipose tissue can be used as a new biomarker to provide a new dimension for renal tumor-related research and may provide new perspectives for personalized tumor management decision-making approaches.

**Key points:**

• There are sex differences in distribution of subcutaneous fat and visceral fat.

• The SFI, VFI, TFI, and rVAT were significantly lower in high-grade ccRCC male patients, but not for female patients.

• Intermuscular fat index can be used as a reliable independent predictor of high-grade ccRCC for both males and females.

**Graphical Abstract:**

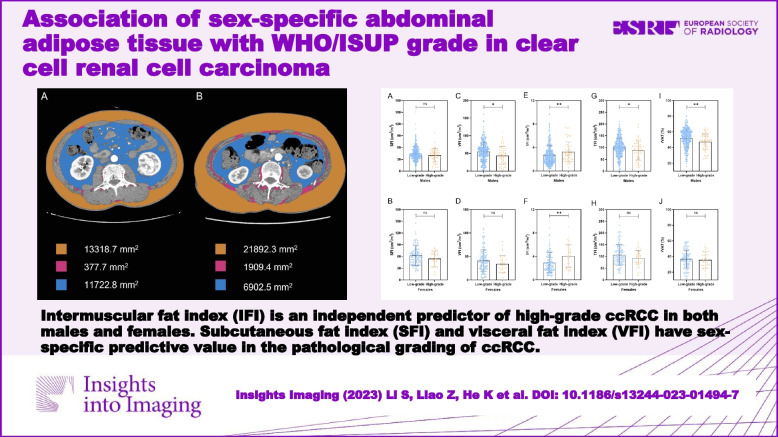

## Introduction

Worldwide, renal cell carcinoma (RCC) ranked as the ninth most frequently diagnosed cancer in men and the 14^th^ in women [1]. Clear cell renal cell carcinoma (ccRCC), the most common histological subtype, originates from proximal tubular cells of the nephron and accounts for approximately 75% of all cases [2]. The incidental renal mass detection rates have increased dramatically in recent decades, possibly because of the widespread use of abdominal imaging [3]. Over the next few decades, the incidence of ccRCC is predicted to increase due to obesity and the aging population [2]. Recently, treatment options for individuals with clinical T1 renal tumors have received considerable attention. Partial nephrectomy preserves more nephrons, but it may lead to a higher likelihood of recurrence and worse prognosis in high-grade tumors [4]. Balancing the competing risks associated with tumors against those related to comorbidities and performance status is essential for treatment decision-making.

Currently, the WHO/ISUP nucleolar grade has been adopted to replace the traditional Furhman grading system in routine clinical practice. The WHO/ISUP grading has been proven to be strongly associated with the prognosis of individuals with ccRCC and papillary RCC [5]. Although renal mass biopsy yields high diagnostic accuracy for discriminating RCC subtypes, the accuracy of pathological grades is much less reliable [6]. Many attempts have been made to preoperatively evaluate the pathological grades of renal tumors, using clinical and radiological modalities.

Obesity is closely associated with kidney cancer. Previous studies have reported that obesity is associated with RCC carcinogenesis and may impair therapeutic effects and surgical outcomes [7, 8]. Although BMI is widely used to assess obesity status, it cannot quantitatively define the distribution of body fat or distinguish between muscle and adipose tissue [9]. Adipose tissue is generally divided into three parts: subcutaneous adipose tissue (SAT), visceral adipose tissue (VAT), and intermuscular adipose tissue (IMAT). There is a differential adipose tissue distribution between males and females [10]. According to Zhu et al., increased VAT is associated with a higher Furhman grade [11], whereas Maurits et al. reported the opposite results [12]. This contradictory result suggests that the relationship between visceral fat and pathological tumor grade requires further investigation. Furthermore, none of these studies focused on the intermuscular fat. Hence, the true effect of adipose tissue on cancer pathology should be further investigated based on each component.

In this study, we attempted to thoroughly explore the association between sex-specific body adipose composition and WHO/ISUP grades of ccRCC using a larger sample size.

## Materials and methods

### Patients

**Fig. 1:**
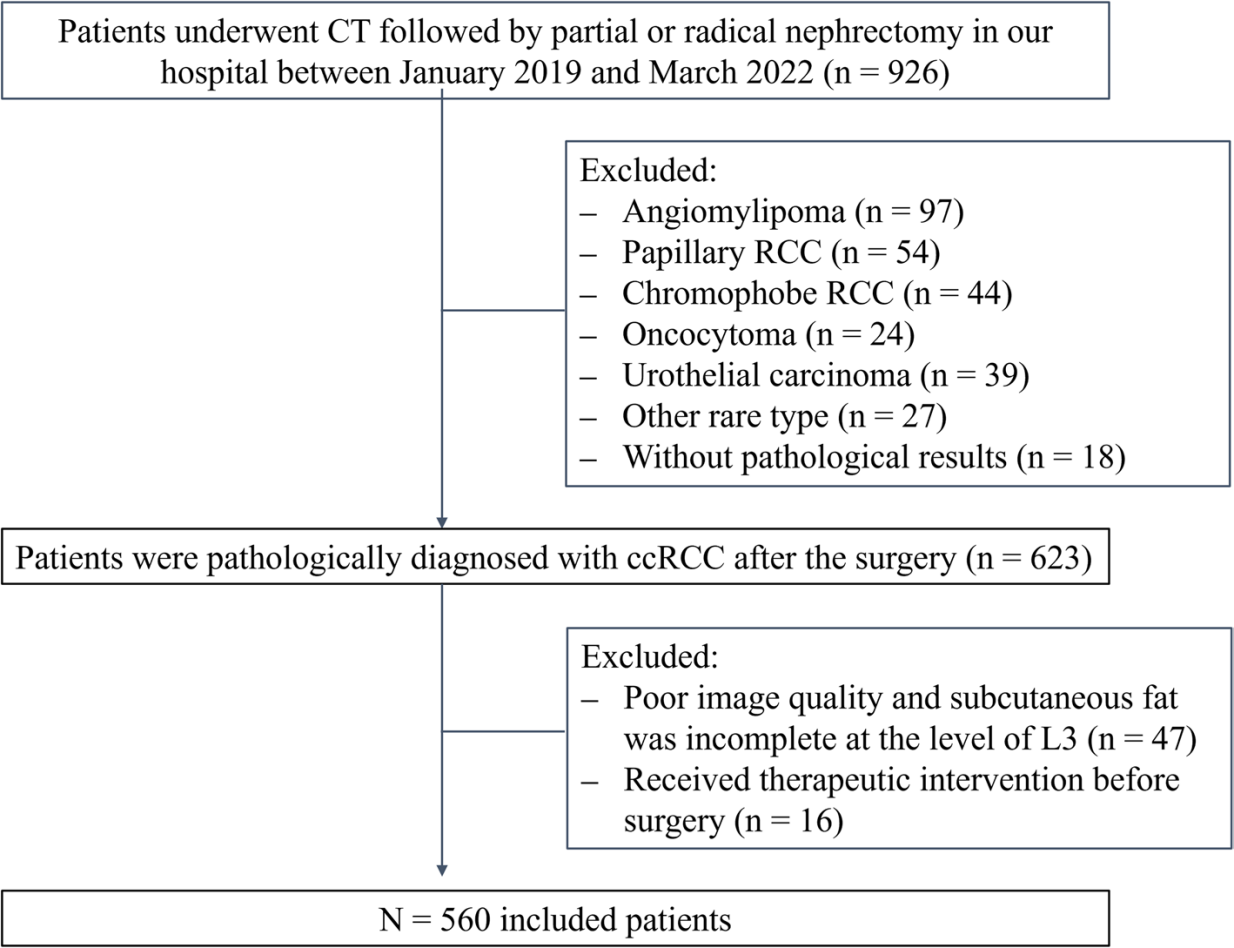
Flowchart of the study population. CT, computed tomography; RCC, renal cell carcinoma. ccRCC, clear cell renal cell carcinoma

This retrospective study adhered to the Declaration of Helsinki's rule of ethics and was approved by our hospital’s ethics committee, and the requirement for informed consent was waived. Data from January 2019 to March 2022 were obtained from the hospital information system, and CT images were downloaded from the Picture Archiving and Communication System. The inclusion criteria were as follows: (1) postoperative pathology confirmed ccRCC with WHO/ISUP grade, and (2) available preoperative CT scans with full axial abdominal CT images. The following were the exclusion criteria: (1) received therapeutic intervention before surgery; (2) abdominal CT images were not fully scanned; and (3) pathological confirmed pathology other than ccRCC. Ultimately, 560 eligible patients (394 males and 166 females) were included in this study (Fig. 1).

### CT anthropometric measurements

All CT scans were performed using one of these scanners (Optima 660, GE Healthcare, Chicago; uCT 780, United Imaging, Shanghai; Somatom Definition AS + , Siemens Healthcare, Erlangen). An intravenous contrast agent (iodixanol 320 mg/mL) was injected at a rate of 3.0–3.5 mL/s (within 20 s), dose dependent on the patient’s weight (1 mL/kg body weight, with a maximum of 150 mL), and was subsequently flushed with 25 mL of saline. After contrast agent administration, the corticomedullary phase was acquired at 25–28 s, and the nephrography phase at 65–70 s. All images were obtained from routine clinical CT scans and there were no defined recommendations for the acquisition and reconstruction protocols. These were the precise details: tube voltage, 120 kV; tube current, varied with dose modulation; matrix, 512 × 512 mm; and reconstructed slice thickness, 0.625–1.250 mm. Images selected from the arterial phase were used for the subsequent measurements.

**Fig. 2:**
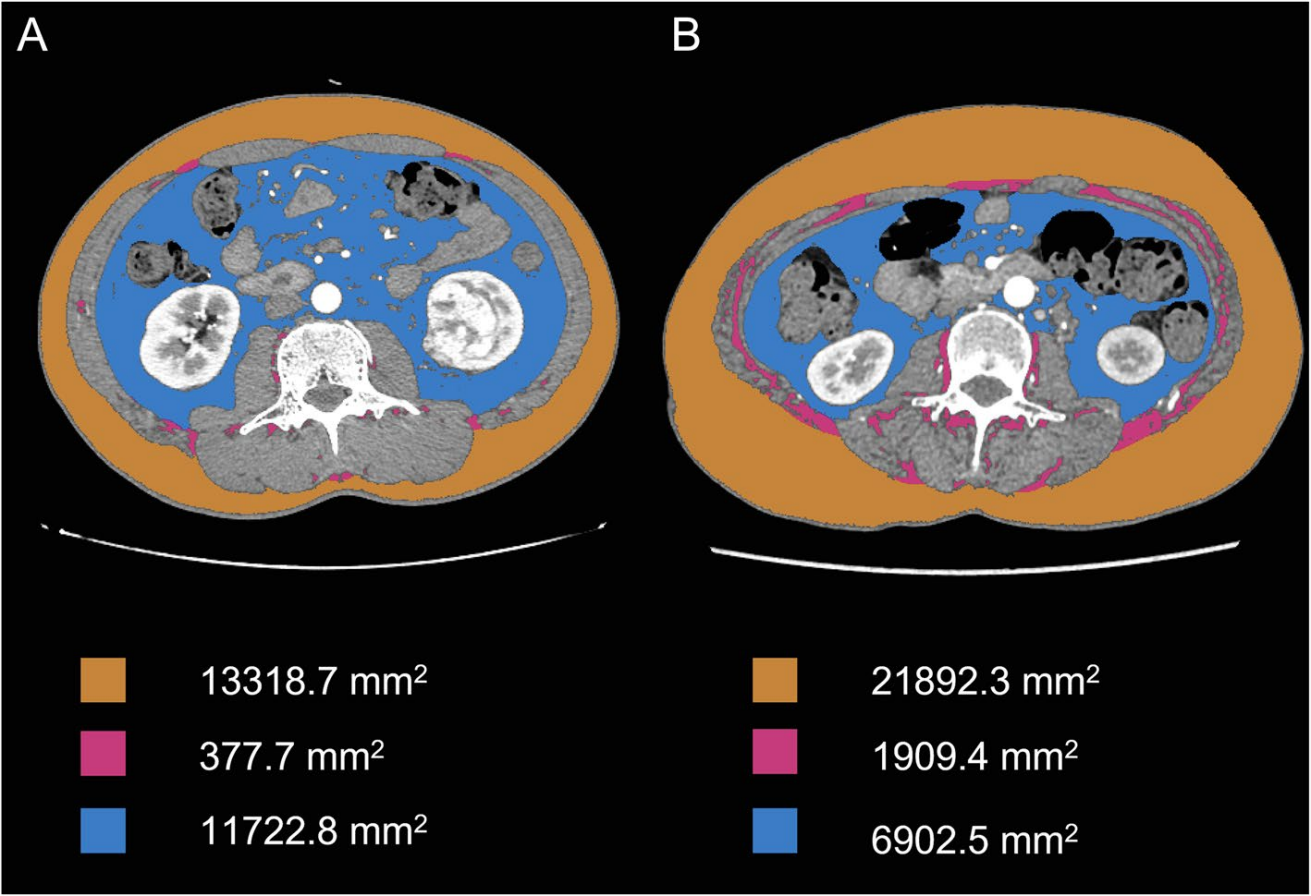
Representative cross-sectional CT images and fat area of each part. **A** Image of a 42-year-old man with WHO/ISUP II ccRCC. **B** Image of a 60-year-old woman with WHO/ISUP IV ccRCC. The subcutaneous adipose tissue area (orange), intramuscular adipose tissue area (red), and visceral adipose tissue area (blue) were quantified

Blinded to the clinical and pathological details of the patients, two trained radiologists (L.Z.Y. and L.S.C., with 2 and 6 years of experience) performed the image analysis. Thirty cases were randomly selected to evaluate the interobserver reproducibility. Independent observer reviewed the images and selected an axial CT slice at the middle level of the L3 vertebra where both pedicles could be seen. First, the total adipose tissue area of the three compartments was semi-automatically calculated using the ImageJ software (National Institutes of Health, USA) based on predefined Hounsfield unit thresholds (− 190 to − 30). The SAT’s outer and inner edges were identified using the wand tool. IMAT was measured after removing all adipose tissue outside the muscle area. Finally, the SAT and IMAT were subtracted from the total adipose tissue, and a clean VAT was obtained by manual adjustment. Further details have been described previously [13]. The distribution of different portions of adipose tissue distribution is shown in Fig. 2. Similar to the BMI, further indices based on the three types of fat area described above were calculated by dividing the fat area by the height of the patient’s height to counteract the impact of body size. Finally, the intermuscular fat index (IFI), subcutaneous fat index (SFI), and visceral fat index (VFI) were subsequently determined. rVAT is the ratio of VAT and TAT.

### WHO/ISUP grade and clinical characteristics

Both clinical and pathological information was collected from electronic medical record system of our hospital. The WHO/ISUP grade was obtained from the postoperative pathological report. And the T-stage was evaluated according to the eighth American Joint Committee on Cancer TNM staging manual. All pathologic results in our hospital were reviewed and reported by experienced senior pathologists.

### Statistical analysis

Data are presented as mean ± standard deviation or number (proportion). We compared categorical data using the chi-square test. Student *t* test or Mann–Whitney *U* test was carried out depending on the distribution, and the Shapiro–Wilk test was employed to examine the normality of continuous data. The association between these variables and a high WHO/ISUP grade of ccRCC was evaluated using univariate logistic regression analysis. For the multivariate analysis, variables with *p* 0.05 were chosen, and odds ratios (ORs) and 95% confidence intervals (CIs) were determined.

Receiver operating characteristic (ROC) curve analysis was used to evaluate the discriminative ability of the different models, and the area under the ROC curve (AUC) was measured. In addition, Spearman’s rank correlation analysis was used to correlate SFI, VFI, TFI, IFI, and BMI. Strong correlations (*r* > 0.75), moderate correlations (*r* > 0.50 and ≤ 0.75), weak correlations (*r* > 0.25 and ≤ 0.50), and no correlations (*r* > 0.25) were all judged to exist.

SPSS (version 26.0, IBM) and GraphPad Prism (version 8.3.0, GraphPad Software Inc., USA) were used for all statistical analyses. A two-tailed significance threshold of *p* < 0.05 was applied.

## Results

### Clinical characteristics

Five hundred sixty patients (374 males and 166 females; mean age, 55.9 ± 10.5 years; age range, 23–85 years; tumor size, 4.6 ± 2.2 cm) were included in this retrospective study. All participants underwent partial or radical nephrectomy and had postoperative pathologically confirmed ccRCC (139 WHO/ISUP grade I lesions, 328 WHO/ISUP grade II lesions, 69 WHO/ISUP grade III lesions, and 24 WHO/ISUP grade IV lesions). The clinicopathological information for the 560 participants is shown in Table 1.

**Table 1:** Baseline characteristics of the population

Variable	*N* = 560
Sex	
Male	394 (70.4%)
Female	166 (29.6%)
Age (years)	55.5 ± 10.9
Size (cm)	4.6 ± 2.2
Side	
Right	256 (45.7%)
Left	304 (54.3%)
WHO/ISUP grade	
I	139 (24.8%)
II	328 (58.6%)
III	69 (12.3%)
IV	24 (4.3%)
Pathological T stage	
T1a	295 (52.7%)
T1b	146 (26.1%)
T2a	52 (9.3%)
T2b	5 (0.9%)
T3a	59 (10.5%)
T3b	2 (0.4%)
T4	1 (0.2%)

Continuous data are expressed as mean ± standard deviation (interquartile range) and categorical data as number (proportion)

*BMI* Body mass index

### Interobserver reliability assessment

The interobserver consistency between the two radiologists was excellent. The intraclass correlation coefficients (ICCs) and 95% confidence intervals for each adipose tissue parameter were as follows: SAT (ICC = 0.999, 95% CI: 0.998–1.000); IMAT (ICC = 0.997, 95% CI: 0.993–0.998); VAT (ICC = 1.000, 95% CI: 1.000–1.000). The results indicate good measurement repeatability for adipose tissue measurements.

### Adiposity measurements of ccRCC patients according to sex

The clinical characteristics and adiposity variables of the sexes are presented in Table 2. Males were significantly taller and heavier than females. The SAT, SFI, and IFI were significantly higher in women, and the BMI, VAT, TAT, VFI, and rVAT were higher in males. The IMAT and TFI showed no differences according to sex.

**Table 2:** Clinical and adipose parameters of ccRCC patients according to sex

Variables	Males (*n* = 394)	Females (*n* = 166)	*p*-value
Age (years)	54.9 ± 11.0	56.8 ± 10.5	0.061
Height (m)	1.7 ± 0.1	1.6 ± 0.1	< 0.001^*^
Body weight (kg)	72.2 ± 10.8	60.1 ± 9.7	< 0.001^*^
Size (cm)	4.4 ± 2.1	5.0 ± 2.4	0.037^*^
BMI (kg/m^2^)	24.8 ± 3.2	23.7 ± 3.7	0.001^*^
SAT (mm^2^)	12,559.2 ± 5238.3	15,383.1 ± 6311.5	< 0.001^*^
VAT (mm^2^)	15,400.8 ± 7736.1	10,014.7 ± 5627.9	< 0.001^*^
IMAT (mm^2^)	810.4 ± 481.8	808.8 ± 471.6	0.984
TAT (mm^2^)	28,770.4 ± 11,573.0	26,206.6 ± 10713.5	0.002^*^
SFI (cm^2^/m^2^)	43.2 ± 17.7	60.6 ± 24.5	< 0.001^*^
VFI (cm^2^/m^2^)	53.1 ± 26.7	39.6 ± 22.4	< 0.001^*^
IFI (cm^2^/m^2^)	2.8 ± 1.6	3.2 ± 1.9	0.012^*^
TFI (cm^2^/m^2^)	99.0 ± 39.4	103.4 ± 42.1	0.680
rVAT (%)	51.2 ± 11.6	36.4 ± 11.2	< 0.001^*^

*BMI* Body mass index, *SAT* Subcutaneous adipose area, *VAT* Visceral adipose tissue, *IMAT* Intermuscular adipose tissue, *TAT* Total adipose tissue, *SFI* Subcutaneous fat index, *VFI* Visceral fat index, *IFI* Intramuscular fat index, *TFI* Total fat index, *rVAT* Relative VFA

^*^Significant results

### Sex-specific abdominal fat measurements in comparison between WHO/ISUP low-and high-grade ccRCC

**Fig. 3:**
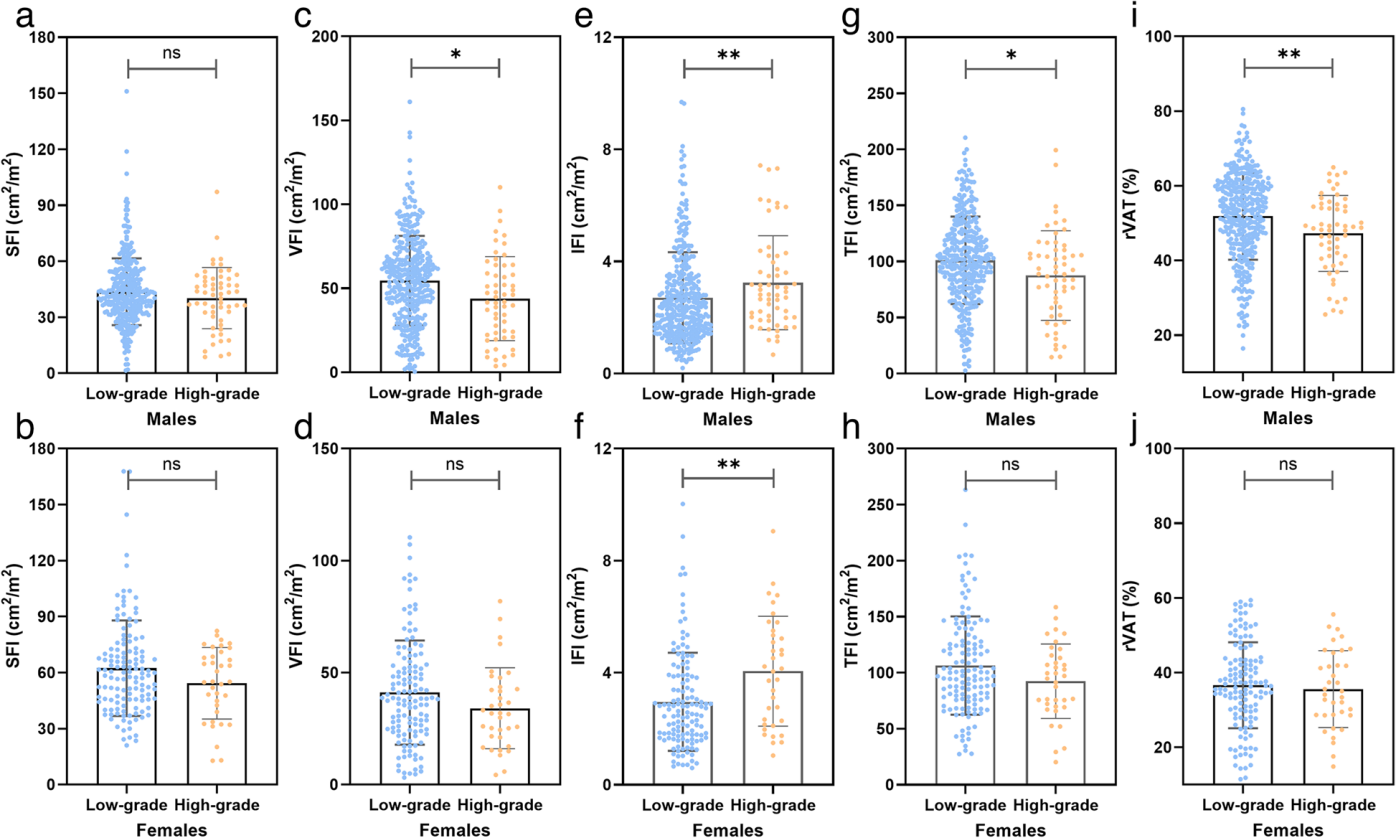
Plots show individual data points by sex for subcutaneous fat index (**a**, **b**), visceral fat index (**c**, **d**), intramuscular fat index (**e**, **f**), total fat index (**g**, **h**), and relative visceral adipose tissue (**i**, **j**) of patients with different pathologic grades

The low- and high-grade groups were compared according to sex (Fig. 3 and Table 3). The SAT and SFI showed no significant difference across the groups in men. In comparison to the low-grade group, the IMAT and IFI were significantly higher, and the BMI, VAT, TAT, VFI, TFI, and rVAT were significantly lower in the high-grade group. In females, the IMAT and IFI were significantly higher, and the TAT was significantly lower in the high-grade group.

**Table 3:** Sex-specific abdominal adipose tissue measurements stratified by WHO/ISUP grade

Variables	Male		*p*-value	Female		*p*-value
	Low-grade (*n* = 337)	High-grade (*n* = 57)		Low-grade (*n* = 91)	High-grade (*n* = 19)	
Age (years)	54.6 ± 10.9	56.9 ± 11.5	0.099	56.1 ± 10.2	59.4 ± 11.6	0.101
Size (cm)	4.1 ± 1.9	6.1 ± 2.6	< 0.001^*^	4.5 ± 2.2	6.6 ± 2.7	< 0.001^*^
BMI (kg/m^2^)	24.9 ± 3.2	23.9 ± 3.1	0.028^*^	23.9 ± 3.8	23.0 ± 3.3	0.194
SAT (mm^2^)	12,716.1 ± 5288.9	11,631.7 ± 4869.2	0.335	15,910.6 ± 6583.7	13,478.0 ± 4823.5	0.133
VAT (mm^2^)	15,862.3 ± 7729.6	12,672.6 ± 7257.4	0.004^*^	10,452.1 ± 5858.6	8434.8 ± 4419.6	0.057
IMAT (mm^2^)	790.0 ± 480.3	930.6 ± 477.3	0.014^*^	754.3 ± 450.3	1005.8 ± 500.3	0.004^*^
TAT (mm^2^)	29,368.4 ± 11,468.7	25,234.9 ± 11,656.3	0.012^*^	27,117.1 ± 11,171.1	22,918.6 ± 8182.4	0.037^*^
SFI (cm^2^/m^2^)	43.7 ± 17.9	40.3 ± 16.4	0.392	62.4 ± 25.6	54.3 ± 19.2	0.297
VFI (cm^2^/m^2^)	54.6 ± 26.6	43.9 ± 25.0	0.004^*^	41.1 ± 23.2	34.1 ± 18.1	0.096
IFI (cm^2^/m^2^)	2.7 ± 1.6	3.2 ± 1.7	0.009^*^	3.0 ± 1.8	4.1 ± 2.0	0.002^*^
TFI (cm^2^/m^2^)	101.0 ± 39.0	87.4 ± 39.9	0.018^*^	106.4 ± 43.9	92.4 ± 33.2	0.174
rVAT (%)	51.9 ± 11.7	47.2 ± 10.2	0.002^*^	36.6 ± 11.5	35.6 ± 10.3	0.629

*BMI* Body mass index, *SAT* Subcutaneous adipose area, *VAT* Visceral adipose tissue, *IMAT* Intermuscular adipose tissue, *TAT* Total adipose tissue, *SFI* Subcutaneous fat index, *VFI* Visceral fat index, *IFI* Intramuscular fat index, *TFI* Total fat index, *rVAT* Relative VFA

^*^Significant results

### High-grade ccRCC prediction using univariate and multivariate logistic regression analysis

Univariate logistic regression analysis was conducted on each variable in both males and females to establish the relationship between sex-specific abdomen fat measurements and the pathological grade of ccRCC. Variables with *p* < 0.1 were further incorporated into the multivariate analysis (Table 4). For males, the multivariate model identified size (OR 1.039, 95% CI 0.025–1.053, *p* < 0.001), VFI (OR 0.979, 95% CI 0.965–0.992, *p* = 0.002), and IFI (OR 1.408, 95% CI 1.162–1.705, *p* < 0.001) as independent significant predictors of high-grade ccRCC. In females, the multivariate model identified size (OR 1.031, 95% CI 1.014–1.049,* p* < 0.001), SFI (OR 0.975, 95% CI 0.955–0.994, *p* = 0.012), and IFI (OR 1.446, 95% CI 1.155–1.810, *p* = 0.001) as significant predictors of high-grade ccRCC.

**Table 4:** Univariate and multivariate logistic regression analysis for predicting high-grade ccRCC

Variables	Univariate analysis-males			Multivariate analysis-males			Univariate analysis-females			Multivariate analysis-females		
	OR	95% CI	*p*-value	OR	95% CI	*p*-value	OR	95% CI	*p*-value	OR	95% CI	*p*-value
Age (years)	1.02	0.994–1.048	0.138				1.032	0.994–1.071	0.103			
Size (cm)	1.04	1.026–1.054	< 0.001^*^	1.039	1.025–1.053	< 0.001^*^	1.035	1.018–1.051	< 0.001^*^	1.031	1.014–1.049	< 0.001^*^
BMI (kg/m^2^)	0.903	0.824–0.990	0.029^*^				0.933	0.840–1.036	0.194			
SFI (cm^2^/m^2^)	0.988	0.971–1.005	0.181				0.984	0.967–1.002	0.080	0.975	0.955–0.994	0.012^*^
VFI (cm^2^/m^2^)	0.984	0.973–0.995	0.005^*^	0.979	0.965–0.992	0.002^*^	0.985	0.967–1.003	0.098			
IFI (cm^2^/m^2^)	1.192	1.021–1.391	0.026^*^	1.408	1.162–1.705	< 0.001^*^	1.345	1.108–1.632	0.003^*^	1.446	1.155–1.810	0.001^*^
TFI (cm^2^/m^2^)	0.991	0.984–0.998	0.017^*^				0.991	0.982–1.001	0.079			
rVAT (%)	0.967	0.944–0.990	0.006^*^				0.992	0.960–1.025	0.627			

*BMI* Body mass index, *SFI* Subcutaneous fat index, *VFI* Visceral fat index, *IFI* Intramuscular fat index, *TFI* Total fat index, *rVAT* Relative VFA

^*^Significant results

**Fig. 4:**
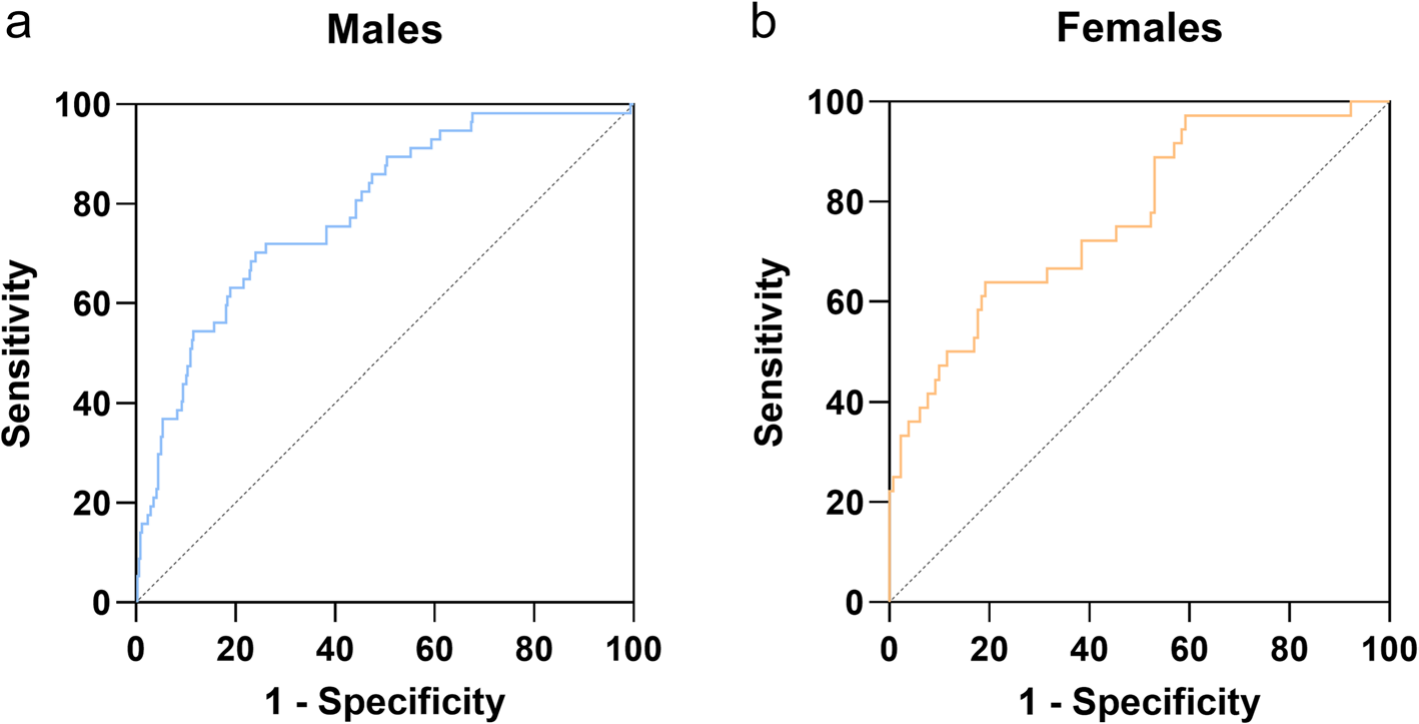
ROC curves and area under the curve (AUC) of WHO/ISUP grading were evaluated for male (**a**) and female (**b**) patients with different models

As shown in Fig. 4, for males, the AUC of the multivariate model was 0.785 (95% CI 0.722–0.848). And for females, the AUC of the multivariate model was 0.769 (95% CI 0.681–0.857).

### Correlation analysis

**Fig. 5:**
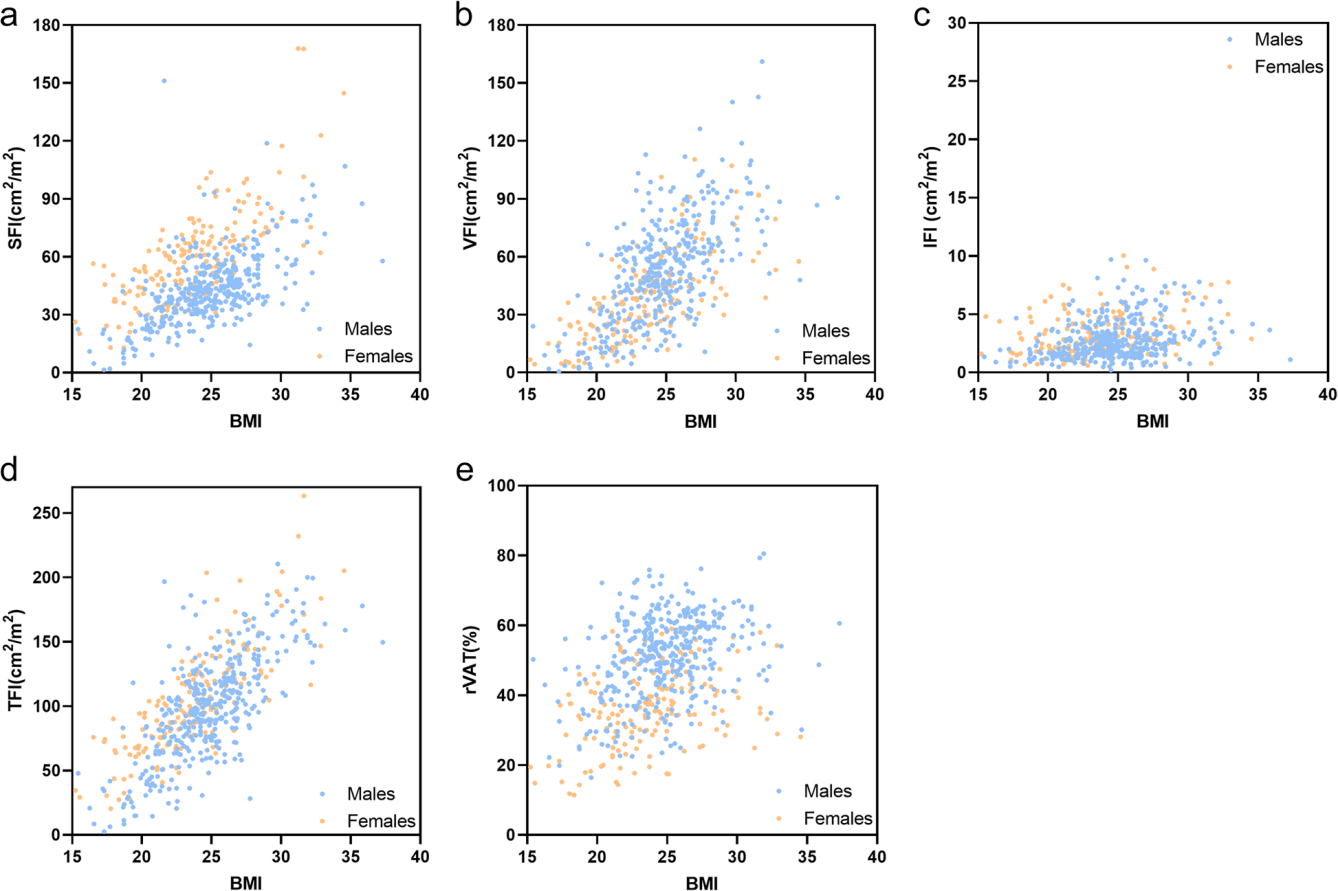
Scatterplots of BMI vs. subcutaneous fat index (**a**), BMI vs. visceral fat index (**b**), BMI vs. intramuscular fat index (**c**), BMI vs. total fat index (**d**), and BMI vs. relative VAT (**e**) according to sex

SFI (males, *r* = 0.596, *p* < 0.001; females, *r* = 0.726, *p* < 0.001) and VFI (males, *r* = 0.604, *p* < 0.001; females, *r* = 0.719, *p* < 0.001) were both moderately positively correlated with BMI. The TFI had a moderate positive correlation with BMI in males (*r* = 0.673, *p* < 0.001) but had a strong positive correlation with BMI in females (*r* = 0.815, *p* < 0.001). The IFI (males, *r* = 0.296, *p* < 0.001; females, *r* = 0.255, *p* < 0.001) and rVAT (males, *r* = 0.305, *p* < 0.001; females, *r* = 0.342, *p* < 0.001) were both weakly positively correlated with BMI (Fig. 5).

## Discussion

In this study of 560 patients with ccRCC, we found that IFI was an independent predictor of high-grade ccRCC in both males and females. Unlike previous studies, this result indicates that intermuscular adipose tissue, rather than visceral fat, is the most valuable component of abdominal fat for the pathological grading of ccRCC. In addition, SFI and VFI have sex-specific prognostic value in the pathological grading of ccRCC.

Epidemiological studies suggest that obesity may be a significant risk factor for kidney cancer [14, 15]. However, there exists a paradox between obesity and improved prognosis. The relationship of obesity to the pathological grade of kidney cancer remains unclear. Consistent with the findings of this study, Hu et al. reported that BMI was not a significant risk factor for high-grade ccRCC. This may be because BMI is a general concept that does not reflect the fat content, and the pathological grade of ccRCCs may be related to specific changes in the composition of one or more fat types.

Our study revealed significant differences in fat components between the sexes. Male patients had significantly higher BMI, VAT, TAT, and rVAT values than female patients, whereas female patients had significantly higher SAT values than male patients. Similar findings have been reported by Park et al. [16], and demonstrated sexual dimorphism based on anthropometric measurements, with males tending to accumulate more visceral fat and females accumulating more fat in the subcutaneous depot [17]. Therefore, consideration of sex in obesity-related studies is highly warranted.

Several studies have reported that visceral fat is associated with the pathological grade of RCC [11, 18, 19]. However, the results of these studies were inconsistent. According to Zhu et al. [11], VAT% (OR 1.06, 95% CI 1.02–1.09, *p* = 0.0018) was an independent prognostic predictor for a higher Furhman grade in patients with cT1a RCC. Hu et al. [19] reported similar findings and found that rVAT (OR 1.06, 95% CI 1.02–1.09, *p* = 0.0018) was the only predictor of pathological grade in female patients with ccRCC. However, Maurits et al. [12] found no association between visceral fat and the pathological grade of RCC. In the present study, VAT, VFI, IMAT, IFI, TAT, TFI, and rVAT were associated with the pathological grade of ccRCC in males, whereas only IMAT, IFI, and TAT were associated with the pathological grade of ccRCC in females. Although these results appear to be contradictory, meaningful conclusions can be drawn. First, there may be considerable heterogeneity by sex. Second, these studies agree that subcutaneous fat content is not related to the pathological grade of renal tumors. Lastly, the study by Maurits et al. [12] was based on a large sample size, whereas the studies by Zhu et al. [11] and Hu et al. [19] had a small sample size; therefore, the association between visceral fat and the pathological grade of kidney tumors may be weak or even unrelated. However, none of these studies calculated adipose tissue index to balance out the effect of body size, nor did they measured intermuscular fat and included it in further analyses.

In this study, we discovered that intermuscular fat was significantly higher, regardless of sex, in patients with high-grade ccRCC. We first identified IFI as an independent predictor of pathological grade in patients with ccRCC. Among the fat components, intermuscular fat has received little attention in cancer-related studies. Previous studies have found different mRNA expression patterns in visceral, subcutaneous, and intermuscular depots, indicating that each fat component represents a unique regulatory tissue [20, 21]. Similar to visceral adipose tissue, increased intermuscular fat is associated with poor physical and metabolic outcomes, including diabetes, chronic kidney disease, and cardiovascular disease [22–25]. Intermuscular fat has recently been shown by Yu et al. [26] to be a significant risk factor for major complications after primary total hip arthroplasty. Jeon et al. [27] reported that the intermuscular fat index is associated with breast cancer prognosis. This may be related to the fact that high-grade tumors promote an increase in intermuscular fat with antioxidant and anti-inflammatory effects. However, the underlying biological mechanism remains unclear. Investigations on human IMAT are essential for further adipose biology research.

In the present study, low VFI and low SFI were independent risk factors for high-grade ccRCC in men and women, respectively. Keehn et al. [18] reported that VAT may be associated with high pathological grade in patients with small renal masses. This is consistent with the results of our study. Visceral fat can secrete adipokines such as leptin and adiponectin [28]. Clinical studies have shown that low adiponectin is associated with tumorigenesis and poor prognosis [29, 30]. And SAT produces leptin and maintains insulin sensitivity, thus impairing tumor progression [31]. These may explain the association of higher VFI and SFI with lower tumor grade.

There are several limitations to this study. First, this was a single-center, retrospective study. Second, we only studied the fat area and did not explore the association between fat density and the pathological grade of ccRCC because the images were not obtained from plain CT scans. Third, this study is the first to suggest an association between intermuscular fat and kidney tumor grade. Larger confirmatory studies are needed to verify and evaluate this association.

In conclusion, intermuscular fat index is a valuable biomarker for the pathological grade of ccRCC and could be used as a reliable independent predictor of high-grade ccRCC for both males and females. Sex-specific differences in adipose tissue distribution contribute to the varying predictive values of ccRCC grades between males and females. This study provides valuable insights into the importance of considering sex-specific adipose tissue components in the pathological grading of ccRCC, potentially paving the way for personalized treatment approaches based on these specific fat depots.

## Data Availability

The datasets used and/or analyzed during the current study are available from the corresponding author on reasonable request.

## Abbreviations

AUC: Area under the curve
BMI: Body mass index
ccRCC: Clear cell renal cell carcinoma
CT: Computed tomography
IFI: Intermuscular fat index
IMAT: Intermuscular adipose tissue
OR: Odds ratio
ROC curve: Receiver operating characteristics curve
rVAT: Relative visceral adipose tissue
SAT: Subcutaneous adipose tissue
SFI: Subcutaneous fat index
TAT: Total adipose index
TFI: Total fat index
VAT: Visceral adipose tissue
VFI: Visceral fat index

## References

[1] Sung H, Ferlay J, Siegel RL (2021). Global Cancer Statistics 2020: GLOBOCAN estimates of incidence and mortality worldwide for 36 cancers in 185 countries. CA Cancer J Clin.

[2] Turajlic S, Swanton C, Boshoff C (2018). Kidney cancer: the next decade. J Exp Med.

[3] Pedrosa I, Cadeddu JA (2022). How we do it: managing the indeterminate renal mass with the MRI clear cell likelihood score. Radiology.

[4] Suzuki K, Mizuno R, Mikami S (2012). Prognostic significance of high nuclear grade in patients with pathologic T1a renal cell carcinoma. Jpn J Clin Oncol.

[5] Paner GP, Stadler WM, Hansel DE, Montironi R, Lin DW, Amin MB (2018). Updates in the eighth edition of the tumor-node-metastasis staging classification for urologic cancers. Eur Urol.

[6] Sanchez A, Feldman AS, Hakimi AA (2018). Current management of small renal masses, including patient selection, renal tumor biopsy, active surveillance, and thermal ablation. J Clin Oncol.

[7] Graff RE, Wilson KM, Sanchez A (2022). Obesity in relation to renal cell carcinoma incidence and survival in three prospective studies. Eur Urol.

[8] Boi SK, Orlandella RM, Gibson JT (2020). Obesity diminishes response to PD-1-based immunotherapies in renal cancer. J Immunother Cancer.

[9] Li S, Qiu R, Yuan G (2022). Body composition in relation to postoperative anastomotic leakage and overall survival in patients with esophageal cancer. Nutrition.

[10] Jeffery E, Wing A, Holtrup B (2016). The adipose tissue microenvironment regulates depot-specific adipogenesis in obesity. Cell Metab.

[11] Zhu Y, Wang HK, Zhang HL (2013). Visceral obesity and risk of high grade disease in clinical t1a renal cell carcinoma. J Urol.

[12] Maurits JSF, Sedelaar JPM, Aben KKH, Kiemeney L, Vrieling A (2022). Association of visceral and subcutaneous adiposity with tumor stage and Fuhrman grade in renal cell carcinoma. Sci Rep.

[13] Gomez-Perez SL, Haus JM, Sheean P (2016). Measuring abdominal circumference and skeletal muscle from a single cross-sectional computed tomography image: a step-by-step guide for clinicians using National Institutes of Health ImageJ. JPEN J Parenter Enteral Nutr.

[14] Petrelli F, Cortellini A, Indini A (2021). Association of obesity with survival outcomes in patients with cancer: a systematic review and meta-analysis. JAMA Netw Open.

[15] Lauby-Secretan B, Scoccianti C, Loomis D, Grosse Y, Bianchini F, Straif K (2016). Body fatness and cancer–viewpoint of the IARC Working Group. N Engl J Med.

[16] Park YH, Lee JK, Kim KM (2014). Visceral obesity in predicting oncologic outcomes of localized renal cell carcinoma. J Urol.

[17] Palmer BF, Clegg DJ (2015). The sexual dimorphism of obesity. Mol Cell Endocrinol.

[18] Keehn A, Srivastava A, Maiman R (2015). The relationship between visceral obesity and the clinicopathologic features of patients with small renal masses. J Endourol.

[19] Hu Z, Wu J, Lai S (2020). Clear cell renal cell carcinoma: the value of sex-specific abdominal visceral fat measured on CT for prediction of Fuhrman nuclear grade. Eur Radiol.

[20] Lee HJ, Park HS, Kim W, Yoon D, Seo S (2014). Comparison of metabolic network between muscle and intramuscular adipose tissues in Hanwoo beef cattle using a systems biology approach. Int J Genomics.

[21] Bong JJ, Cho KK, Baik M (2009). Comparison of gene expression profiling between bovine subcutaneous and intramuscular adipose tissues by serial analysis of gene expression. Cell Biol Int.

[22] Waters DL, Aguirre L, Gurney B (2022). Effect of aerobic or resistance exercise, or both, on intermuscular and visceral fat and physical and metabolic function in older adults with obesity while dieting. J Gerontol A Biol Sci Med Sci.

[23] Koster A, Stenholm S, Alley DE (2010). Body fat distribution and inflammation among obese older adults with and without metabolic syndrome. Obesity (Silver Spring).

[24] Tuttle LJ, Sinacore DR, Mueller MJ (2012). Intermuscular adipose tissue is muscle specific and associated with poor functional performance. J Aging Res.

[25] Madero M, Katz R, Murphy R (2017). Comparison between different measures of body fat with kidney function decline and incident CKD. Clin J Am Soc Nephrol.

[26] Yu J, Wang M, Shen H (2022) Intermuscular fat, but not subcutaneous fat, correlated with major complications after primary total hip arthroplasty. Acad Radiol 10.1016/j.acra.2022.09.014 10.1016/j.acra.2022.09.014 36253236

[27] Jeon YW, Park HS, Ko Y (2021). Intermuscular fat density as a novel prognostic factor in breast cancer patients treated with adjuvant chemotherapy. Breast Cancer Res Treat.

[28] Aparecida Silveira E, Vaseghi G, de Carvalho Santos AS (2020). Visceral obesity and its shared role in cancer and cardiovascular disease: a scoping review of the pathophysiology and pharmacological treatments. Int J Mol Sci.

[29] Muppala S, Konduru SKP, Merchant N (2017). Adiponectin: Its role in obesity-associated colon and prostate cancers. Crit Rev Oncol Hematol.

[30] Mistry T, Digby JE, Desai KM, Randeva HS (2007). Obesity and prostate cancer: a role for adipokines. Eur Urol.

[31] Kim JM, Chung E, Cho ES (2021). Impact of subcutaneous and visceral fat adiposity in patients with colorectal cancer. Clin Nutr.

